# A community Legionnaires’ disease outbreak linked to a collective biomass condensing boiler, France, 2019

**DOI:** 10.2807/1560-7917.ES.2025.30.41.2400804

**Published:** 2025-10-16

**Authors:** Sophie Raguet, Christophe Ginevra, Ghislaine Descours, Clémence Augustin, Astrid Rebert-Placide, Michel Vernay, Sophie Jarraud, Christine Campèse

**Affiliations:** 1 The French Public Health Agency, Grand Est, Strasbourg, France; 2 Hospice Civils de Lyon, National Reference Center of Legionella, Lyon, France; 3 CIRI Centre International de Recherche en Infectiologie, Legiopath team, Inserm, U1111, CNRS, UMR5308, Ecole Normale Supérieure de Lyon, Université Claude Bernard Lyon 1, Lyon, France; 4 ESCMID Study Group for Legionella Infections (ESGLI), Basel, Switzerland; 5 GenEPII Sequencing Platform, Institut des Agents Infectieux, Hospices Civils de Lyon, Lyon, France; 6 Regional Health Authorities, Grand Est, Strasbourg, France; 7 The French Public Health Agency, Saint Maurice, France

**Keywords:** Legionella, Legionnaires’ disease, outbreak, France, unusual source

## Abstract

Between 1 November and 12 December 2019, a Legionnaires’ disease (LD) outbreak occurred in the Strasbourg metropolitan area, France. Epidemiological, environmental and genomic investigations (nested sequence-based typing and whole genome sequencing (WGS)) were undertaken to locate the outbreak source, characterise the causal *Legionella* strain, and understand its spread. Through a positive urinary antigen test, 28 cases (14 male; 14 female) with 70 years median age (range: 42–88 years) were diagnosed. Two died. For nine cases, typing revealed *L. pneumophila* serogroup 1 (Lp 1) sequence type (ST) 62 infection. Mapping where cases were during their incubation period pointed to a place south-west of Strasbourg city as the outbreak epicentre. There, in the biomass condensing boiler of a heating plant, high Lp 1 contamination levels (10^5^–10^6^ CFU/L) were discovered. Eight environmental Lp 1 isolates from inside the plant were ST62. Analysing WGS data from these isolates and the nine Lp 1 ST62 clinical isolates, found their sequences clustering with zero to two single nt polymorphisms. Sub-optimal maintenance and warm weather before the boiler started operating may have allowed Lp 1 proliferation within, with boiler fumes subsequently disseminating Lp 1, thereby exposing people. This outbreak highlights potential LD risks arising from innovative energy-saving heat production processes designed to reduce greenhouse gas emissions.

**Figure:**
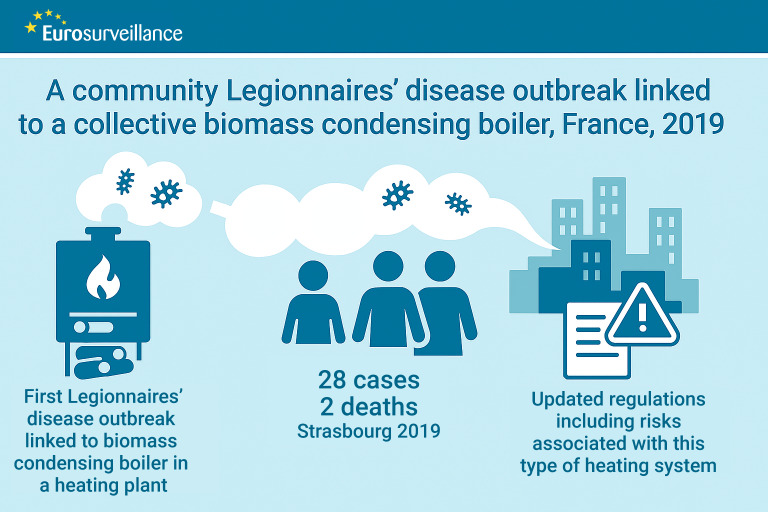

**Key public health message**

**What did you want to address in this study and why?**

Artificial water environments found in showers, cooling towers, whirlpool spas and decorative fountains can allow *Legionella* bacteria to grow. Inhaling aerosolised water contaminated with *Legionella* can lead to pneumonia (i.e. Legionnaires’ disease). In November 2019, several people who had attended an area of metropolitan Strasbourg, France, got Legionnaires’ disease. We aimed to identify the source of *Legionella* bacteria to control this outbreak.

**What have we learnt from this study?**

The outbreak comprised 28 cases of median age 70 years, who became ill between 1 November and 12 December 2019. We identified a boiler in a heating plant as the source. The boiler, which employed a novel energy saving process (energy recovery by condensation), represents a new type of *Legionella* source. Its contamination by *Legionella* bacteria may have resulted from sub-optimal maintenance, as well as the hot summer preceding the outbreak.

**What are the implications of your findings for public health?**

With an aging European population becoming increasingly susceptible to *Legionella* bacteria and climate change leading to a broader adoption of certain energy-saving heating systems that may be conducive to these *Legionella* bacteria, it is important that investigators of Legionnaires’ disease clusters consider such systems as potential outbreak sources.

## Background

Legionnaires’ disease (LD) is a severe pneumonia caused by *Legionella* bacteria. The principal species responsible for LD is *L. pneumophila* of most commonly serogroup 1 (Lp 1), which, in 2021, accounted for 89% of reported cases in Europe [1,2]. Under favourable conditions, such as temperature between 25 and 42 °C, optimum pH, or water hardness, *Legionellae* can live in a wide range of damp and wet environments, including fresh water, soil, sludge and compost. In these settings, protozoa can act as protective hosts, playing an important role in the survival and development of the bacteria within the complex ecosystems of biofilms. Human infections with *Legionella* occur predominantly, but not exclusively, through inhalation of contaminated aerosolised water [1,3,4].

Annual incidence of LD appears to increase with warmer and wetter weather, as well as with higher relative humidity [5-8]. To date, the main sources of *Legionella* contamination identified in outbreaks have been water-cooling towers (WCTs), domestic water systems, spas, decorative fountains, humidifiers, and wastewater treatment plants (WWTPs) [9-14]. Under supportive environmental parameters for their survival, *Legionella* bacteria have been documented to spread in the atmosphere across long distances – over 6 km around a WCT  [15]. As a public health issue, LD can be linked to innovative industrial processes involving the production of water aerosols at a mild temperature (25 –42°C) [16,17].

In France, LD is mandatorily notifiable and regional health authorities conduct systematic investigations of LD cases to identify outbreaks and potential *Legionella* contamination sources. In 2016, the national notification rate was 1.8 cases per 100,000 inhabitants, appearing to further raise, in 2019, to 2.7 cases per 100,000 inhabitants. In 2019, at least one exposure risk during the incubation period of the disease (1 to 10 days before the onset of symptoms) was reported for 39% of cases, pointing to the most frequent risks as travelling (18%), and visiting or staying in hospital settings (6%) or long-term care facilities (5%) [18]. Increased epidemiological surveillance, various guidelines and several regulations have helped to limit the size of outbreaks in France since 2006 [18,19], and between 2006 and 2018, the largest LD outbreak, which occurred in 2017, comprised 18 cases and was linked to an aquatic therapy centre [20].

### Outbreak detection

On 13 November 2019, a hospital in Strasbourg notified four cases of LD with dates of symptom onset ranging from 1 to 9 November. Because all four cases lived in or had visited the same 5-km diameter area of metropolitan Strasbourg (Grand Est region, France) during their incubation period, a common source of contamination was suspected. As per French recommendations, a team including local surveillance partners undertook an environmental investigation to identify the source of infections and to implement control measures [19]. Other cases were subsequently detected, with the outbreak ending on 12 December 2019.

The objective of the present report is to describe the outbreak, which turned out to be caused by a previously unrecognised source of *Legionella* contamination: an innovative energy-saving heating process. We also describe related investigations, with particular emphasis on microbiological and molecular analyses, which not only confirmed the source, but also suggested this source as that of a previously detected outbreak in January 2019.

## Methods

### Epidemiological investigations and case definition

Socio-demographic data, risk factors and clinical data of LD cases were collected by the healthcare workers who diagnosed and notified them, using the standardised mandatory notification questionnaire. Data on sex were collected using a binary variable; the questionnaire is available online: https://www.formulaires.service-public.fr/gf/cerfa_12202_02.do [19].

An outbreak of LD is defined as the occurrence of two or more cases within a specific time and space, potentially involving a common source of contamination [21]. For the outbreak detected in metropolitan Strasbourg in November 2019, a confirmed case was defined as a person with pneumonia and laboratory evidence of *Legionella* infection according to mandatory notification criteria [19], and who had symptom onset between 31 October and 20 December 2019, and who had lived in or visited the Strasbourg area in the 14 days before illness onset.

For all diagnosed cases, physicians were asked to collect lower respiratory tract specimens for *Legionella* culture. A retrospective search of cases was carried out in hospital and private laboratories in the Strasbourg metropolitan area. Relevant people in these establishments were informed of the outbreak to improve the prospective reporting of new cases and for them to send lower respiratory tract specimens to the National Reference Centre of Legionella (NRC-L) in Lyon. General practitioners and hospitals were informed of the alert to minimise diagnostic delays.

The Regional Health Authority covering the metropolitan Strasbourg area interviewed all confirmed cases using a standardised questionnaire that collected data on locations visited in the previous 14 days. We performed a descriptive analysis of the cases and locations visited to identify a possible common contaminated source of infection.

### Environmental investigations

The Regional Health Authority, the Regional Directorate for the Environment, and the French Public Health Agency (*Santé publique France*) jointly investigated contamination in all potential sources of the cases, including individual and common sources. Water samples were taken from 39 potential sources of infection; a total of 83 samples were analysed for the detection and enumeration of *Legionella* according to the French standard NF T90–431:2017 (similar to ISO 11731–2) culture method. A risk analysis of one of the suspected sources – specifically, a biomass condensing boiler – was carried out by a specialist engineering company at the request of the Ministry of the Environment [22].

### Genomic investigations

All the genomic investigations were performed at the NRC-L. Clinical and environmental Lp isolates were typed by whole genome sequencing (WGS), which was performed using a Nextera XT DNA Library Prep Kit (Illumina, San Diego, United States (US)), and paired end 2 × 300 bp on a MiSeq system (Illumina). Genomes were assembled with an in-house pipeline using Trimmomatic, kmerGenie and SPAdes software. Sequence types (ST) were determined based on WGS data using the mompS tool pipeline. One environmental isolate (sample 1, number E-01) was also sequenced using nanopore technology. A sequencing library was prepared using the rapid barcoding kit and sequenced on a Minion R9.4.1 flowcell. An Illumina/nanopore hybrid assembly was performed for this isolate using Unicycler software [23]. This assembly was used as a reference for mapping the Illumina reads of all the other isolates using snippy (https://github.com/tseemann/snippy). Snippy core was used to define the core single nucleotide polymorphisms (SNPs) of the dataset, these core SNPs were used to construct a maximum likelihood phylogenetic tree with FastTree using the general time-reversible and categories (GTR+CAT) model.

Nested-PCR based sequence-based typing (SBT) was performed on culture negative respiratory samples as described previously [24].

## Results

### Outbreak description

**Figure 1:**
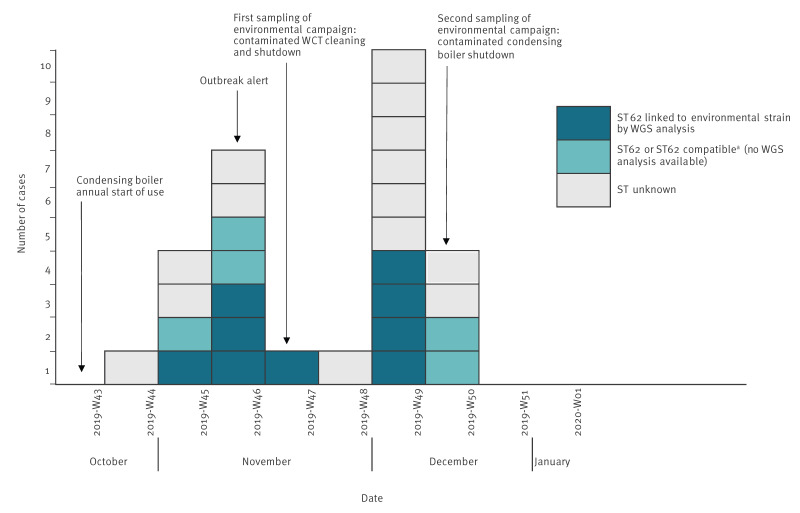
Cases of a Legionnaires’ disease outbreak by date of symptom onset, Strasbourg metropolitan area, France, October–December 2019 (n = 28 cases)

ST: sequence type; W: week; WCT: water-cooling towers; WGS: whole genome sequencing.

^a^ Of the seven genes that characterise ST62, those which could be identified belonged to this ST.

The outbreak comprised 28 cases – all confirmed by a positive Lp 1 urinary antigen test – with illness onset ranging from 1 November to 12 December 2019 (Figure 1). Fourteen cases were males and 14 females, and the median age of the 28 cases was 70 years (range: 42–88 years). Twenty-six cases were hospitalised, 25 had at least one related clinical risk factor such as immunosuppression (i.e. cancer, blood disease, immunosuppressive therapy), diabetes, and smoking. Two cases died. The shape of the epidemic curve was characterised by two peaks: one with seven cases from 11 to 17 November (week 46) and a second with 10 cases from 2 to 8 December (week 49) (Figure 1).

Cases are presented by date of symptom onset. In the legend, for the dark blue square, the ‘environmental strain’ refers to a strain found inside the condensing boiler, which was identified by WGS as the source of the outbreak.

The NRC-L received material for 18 cases including a strain for nine cases and a DNA extract or clinical samples for nine cases. The nine available strains were all of Lp 1 sequence type (ST) 62 as determined by typing. From the nine DNA extracts/clinical samples, no clear ST information could be acquired for four cases (Table). For the five clinical samples remaining, nested-PCR performed directly on the respiratory samples identified all seven genes of ST62 for three cases and some of the seven ST62 genes for the two other cases (described as ‘ST62 compatible’ in the Table). Overall, we had microbiological results for 14 of the 28 cases and no microbiological results for the other 14, either because of no material (i.e. clinical sample, strain, or DNA extract; n = 10), or because the analyses carried out did not provide a clear result (n = 4).

**Table:** Epidemiological and microbiological characteristics of cases of a Legionnaires’ disease outbreak, Strasbourg metropolitan area, France, November–December 2019 (n = 28 cases)

Case number	Year-week of illness	Place of residence or visited	*Legionella pneumophila* 1 urinary antigen testing	ST subtyping	Linked by WGS with environmental strains from the outbreak source	Other environmental contamination identified
1	2019-w45	West district of Strasbourg	( + )	ST62	( + )	No
2	2019-w50	Eckbolsheim	( + )	nd	nd	No
3	2019-w49	West district of Strasbourg	( + )	nd	nd	No
4	2019-w46	West district of Strasbourg	( + )	ST62	( + )	No
5	2019-w46	Strasbourg centre	( + )	ST62	( + )	No
6	2019-w44	West district of Strasbourg	( + )	nd	nd	Yes (home water network – Lp 1)
7	2019-w49	West district of Strasbourg	( + )	nd	nd	No
8	2019-w49	West district of Strasbourg	( + )	nd	nd	No
9	2019-w46	Ostwald	( + )	nd	nd	No
10	2019-w47	West district of Strasbourg	( + )	ST62	( + )	No
11	2019-w49	Strasbourg centre	( + )	nd	nd	Yes (home water network – Lp 2)
12	2019-w49	West district of Strasbourg	( + )	ST62	( + )	No
13	2019-w49	West district of Strasbourg	( + )	ST62	( + )	No
14	2019-w50	Lingolsheim	( + )	nd	nd	No
15	2019-w49	West district of Strasbourg	( + )	ST62	( + )	No
16	2019-w46	Lingolsheim	( + )	ST62	( + )	No
17	2019-w46	Eckbolsheim	( + )	ST62	nd	No
18	2019-w48	West district of Strasbourg	( + )	nd	nd	No
19	2019-w45	West district of Strasbourg	( + )	nd	nd	No
20	2019-w50	Ostwald	( + )	ST62 compatible	nd	No
21	2019-w45	Lingolsheim	( + )	ST62	nd	No
22	2019-w50	West district of Strasbourg	( + )	ST62	nd	No
23	2019-w49	Achenheim	( + )	ST62	( + )	No
24	2019-w46	Lingolsheim	( + )	nd	nd	No
25	2019-w45	West district of Strasbourg	( + )	nd	nd	No
26	2019-w49	Lingolsheim	( + )	nd	nd	No
27	2019-w49	Strasbourg centre	( + )	nd	nd	No
28	2019-w46	West district of Strasbourg	( + )	ST62 compatible	nd	Yes (interfering flora in the home water network)

Lp 1: *Legionella pneumophila* serotype 1; Lp 2: *Legionella pneumophila* serotype 2; nd: non-determined; ST: sequence type; WGS: whole genome sequencing.

**Figure 2:**
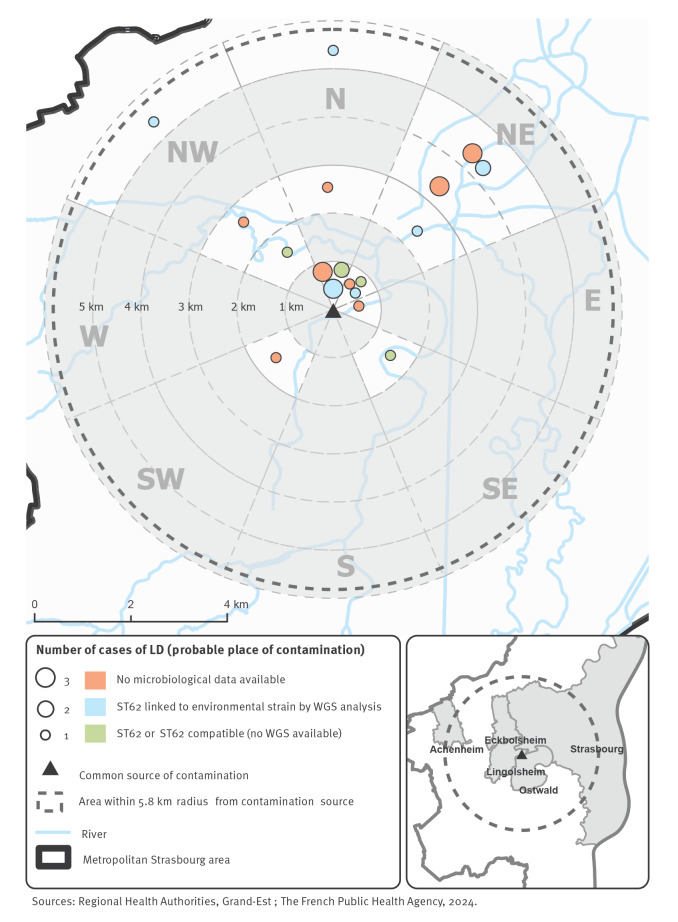
Mapping of cases of a Legionnaires’ disease outbreak, by place attended during the incubation period and microbiological status, Strasbourg metropolitan area, November–December 2019 (n = 28 cases)

At some point during their incubation period, all 28 cases had lived or stayed in a radius of less than 6 km from a place in the north proximity of Lingolsheim, a town located in south-west metropolitan Strasbourg (Figure 2). This radius covered several other towns as well as the west and centre of Strasbourg city. The distribution of cases quickly suggested that the epicentre of the outbreak was in a sector straddling the town of Lingolsheim and Strasbourg city (Figure 2).

E: east; LD: Legionnaires’ disease; N: north; NE: north-east; NW: north-west; S: south; SE: south-east; ST: sequence type; SW: south-west; W: west; WGS: whole genome sequencing.

### Outbreak source investigation

#### Domestic hot water systems

During the investigation of each case water samples were taken from the homes of 14 of the 28 cases to test for *Legionella* contamination. Of these, samples from two homes were contaminated with Lp serogroups 2–15 (one home with a maximum concentration of 10,000 CFU/L, the other 100 CFU/L). The environmental strains were therefore different from the clinical strains (Table). Control measures were immediately implemented (such as thermal shocks or chemical shocks depending on the material composition of the water networks, and descaling).

#### Water-cooling towers and other potential sources

On 20 November 2019 (week 47), an initial sampling campaign was carried out at five companies that operated a WCT located in a 7-km radius of the epicentre (i.e. slightly north of Lingolsheim). No Lp 1 contamination was detected but two WCT were shut down for disinfection due to *Legionella* (i.e. other than Lp 1) contamination.

**Figure 3:**
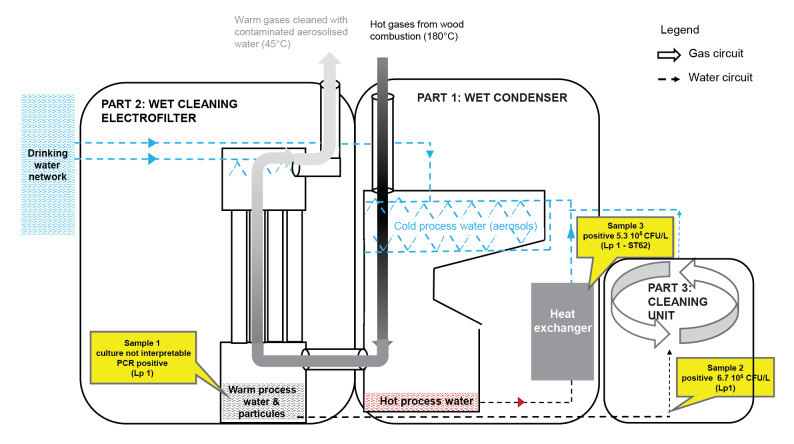
Operating diagram of the biomass condensing heating boiler, which was identified as a Legionnaires’ disease outbreak source, Strasbourg metropolitan area, France, 2019

Source: KoSamti [22].

CFU: colony forming unit; Lp 1: *Legionella pneumophila* serogroup 1; PCR: polymerase chain.

Following the occurrence of new cases, however, a second sampling campaign was carried out on 10 and 11 December (week 50). This second sampling involved 17 companies over a wider area of 10 km, including the five already inspected (see above). All WCT were cleaned without waiting for results. All surveillance samples tested negative for Lp 1. Also, at this time other possible sources in the epicentre were examined, combining field investigations with aerial photo analyses. Several facilities were identified as possible common sources including three car wash stations (1 sample each), four water sprayers for vegetables in supermarkets (1 sample each), and a collective heating plant (3 samples). All these facilities were investigated and, as a precautionary measure, were shut down pending microbiological results. Only the three samples taken from the process water of a biomass condensing boiler in the collective heating plant, showed high levels of Lp contamination (10^5^–10^6^ CFU/L) (samples 1 to 3 in Figure 3).

#### Description and risk assessment of the biomass-condensing boiler

The biomass-condensing boiler was one of four heat sources for a heating plant supplying a newly built neighbourhood. First commissioned in November 2017, the plant location was in the middle of the outbreak epicentre. After being shut down in May 2019 for the summer season, it was restarted on 24 October 2019 (week 43). The plant’s biomass boiler used an innovative energy-saving process, developed with sustainability and climate change in mind: it burned local raw materials (mainly pellets) that are not or cannot be dried as an alternative to fossil fuel. The innovative water-heating process comprised three steps (Figure 3) as follows.

Step one: In the wet condenser, gases at 180 °C from wood pellet combustion were cooled by direct contact with cold tap water. This generated aerosols due to direct contact between fumes and cold water. This heat exchange reduced flue gas temperature from 180 °C to 45 °C and heated the process water. The hot process water was collected, and its energy was recovered by a heat exchanger before the water was reused in the condenser. Sample number 3 was taken at the condenser and tested positive for Lp 1 – ST62 (5.3 10^5^ CFU/L).

Step two: The wet cleaning electrostatic electrofilter purified the fumes from fine particles, before their exited from the chimney. In this process, the cooled flue gases, saturated with water and particles, passed through the electrodes of the electrofilter, which were shaped as vertical tubes. A high voltage current applied to these tubes made them electrostatically charged, allowing their surfaces to attract the fine particles, thereby removing them from the fumes. The precipitator’s surfaces were cleaned with tap water which was dispersed in the air stream. The resulting liquid effluent was collected in a tank and sent to the cleaning unit. Sample number 1 was taken at this cleaning unit and, while its culture results were uninterpretable (because of culture contamination by interfering flora), the sample tested positive for Lp 1 by PCR. Flue gas purification was the last step before the release of the remaining fine particles and aerosols into the atmosphere at 45 °C.

**Figure 4:**
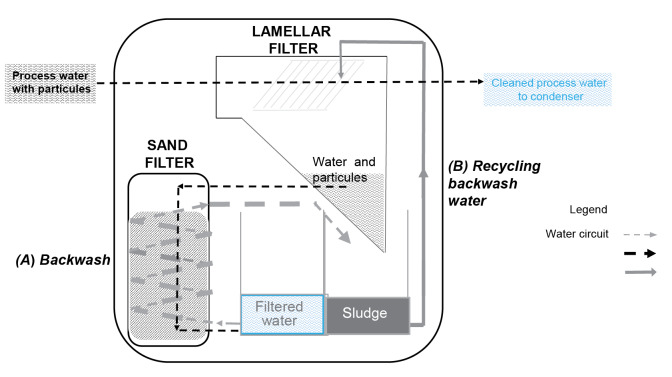
Operating diagram of the cleaning unit of the biomass condensing heating boiler Strasbourg metropolitan area, France, 2019

Source: KoSamti [22].

Step three: For environmental and economic reasons, process water coming from the wet condenser (Part 1 in Figure 3) and the wet cleaning electrofilter (Part 2 in Figure 3) was reused after being cleaned of particles in the cleaning unit (Part 3 in Figure 3). Sample number 2 was taken at the level of the water return to the cleaning unit, and a positive Lp culture was found (6.7 10^5^ CFU/L). The cleaning unit was composed of two filters: a lamellar filter where particles were separated by gravity and a sand filter (Figure 4). Due to the large number of particles, the sand filter frequently clogged, and needed to be washed. This was provided for in the system design, with backwash water from the lamellar filter ((A) in Figure 4) used to clean the sand, and this water was thereafter sent back to the lamellar filter ((B) in Figure 4). Sludge was stored outside in big bags after drying.

This Figure shows more detail about the ‘part 3’ depicted in Figure 3.

Several critical points were found through the risk assessment of the biomass condensing boiler [22]: first, *Legionella* proliferation probably occurred in the cleaning unit as it can grow in sand filters, which can provide a favourable environment for protozoa. The system’s sand filter was not emptied during the summer of 2019, which was particularly hot, fostering *Legionella* multiplication [25]. This led to contamination of the backwash water. Second, recycling backwash water in the lamellar filter led to contamination of the process water. This contamination may also have occurred because of deficiencies in monitoring and recording certain parameters of water quality (e.g. pH, conductivity, microbiological parameters). Third, contaminated process water was in direct contact with fumes, generating a plume saturated with process water at 45 °C and released into the air through the chimneys [26]. This release occurred in a densely populated urban area. Moreover, during the outbreak period, winds predominantly came from the south/south-west, possibly blowing contaminated fumes towards the city of Strasbourg and the affected towns (Supplementary Figure S1); episodes of fog, that can trap and condense the fumes locally, also occurred in this timeframe (field observations).

#### Microbiological and molecular investigations

**Figure 5:**
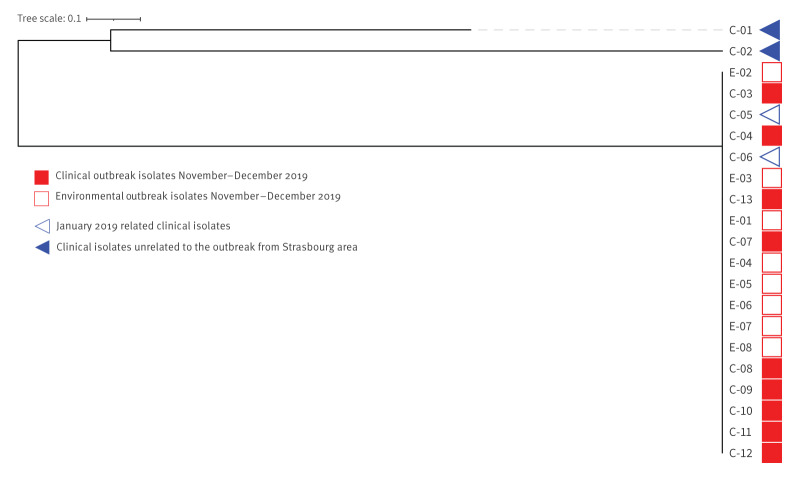
Phylogenetic analysis of *Legionella pneumophila* serogroup 1 ST62 sequences, LD outbreak in Strasbourg metropolitan area, France, November–December 2019 (n = 21 sequences)

Eight environmental isolates of Lp 1 taken in December 2019 from the boiler at the biomass heating plant belonged to ST62. WGS analyses showed that the sequences of the eight isolates clustered with those of the nine clinical isolates with 0 to 2 SNPs (Figure 5). Cases with ST linked to the ST of the environmental isolates were recorded up to 5.8 km from the source (Figure 2). In addition, from the national ST database, the NRC-L retrospectively identified four clinical ST62 isolates among LD cases who were present in the Strasbourg area since 2016. Two of these were not linked to the December 2019 environmental isolates from the heating plant using WGS (> 6,000 SNPs). The two other isolates were linked to the heating plant isolates using WGS (1 SNP for both isolates) (Figure 5). More specifically, the respective two cases were part of a cluster of four cases detected during the previous winter in January 2019. These cases were living or had stayed in the west of metropolitan Strasbourg, within 0.4 km and 5.5 km of the biomass condensing boiler to the north. Investigations into this cluster had failed to identify a common source of contamination. The biomass heating plant, shut down on 12 December (week 50) 2019 for safety reasons, was restarted in October 2020. Since this date, no clinical ST62 isolate has been identified by the NRC-L from diagnosed LD cases living or temporarily staying within 6 km from the boiler.

SNP: single nucleotide polymorphism.

The tree scale indicates the branch length representing 0.1 number of SNP substitutions per site.

## Discussion

All the epidemiological, environmental and microbiological data collected during the investigations pointed to the collective biomass condensing boiler as the common source of contamination of the LD outbreak in metropolitan Strasbourg in 2019. The first cases were identified 10 days after the boiler had been turned on for the 2019 winter season, and no new case occurred after it was shut down in December 2019. The boiler was located at the epicentre of the outbreak, and the clinical and environmental strains were linked using WGS.

Case number 23 was formally linked to the environmental source through WGS analyses, even though the closest this case had come to the contamination source was the village of Achenheim, which is located 5.8 km away. Although recall bias cannot be excluded regarding the places visited, the fact that this case came no closer than Achenheim suggests that contaminated aerosols were dispersed up to 6 km. This distance is shorter than the 12 km-spread reported for other outbreaks associated with WCT [15,27], and the 5 to 10 km reported in a case–control study which suggested direct transmission from the aeration pond of a WWTP [28]. The meteorological conditions during December 2019 in metropolitan Strasbourg (wind, fog) may also have contributed to the spread of *Legionella* over this relatively long distance [5,29], as illustrated in Supplementary Figure S1.

Boilers have not been previously identified as *Legionella* contamination sources, as the usual heating processes involved typically produce high-temperature exhaust gases. In the 2019 Strasbourg outbreak, the boiler in the biomass heating plant was suspected following a field visit by the investigation team based on descriptive epidemiological findings and the strong spatiotemporal clustering of cases. First, the team carried out environmental sampling of the boiler water process. A subsequent detailed risk analysis identified four possible main causes of contamination as follows [22]: (i) insufficient maintenance of the sand filter used to clean the condensate water, leading to the development of protozoa and biofilms; (ii) recycling of the backwash water from the sand filter into the process water, resulting in contamination of the process water; (iii) the generation of a large amount of aerosols from a cooling process where fumes came in contact with process water; (iv) the restarting of the plant after a period of inactivity, which favoured the release of the existing biofilm. To the best of our knowledge, this is the first time that a boiler has ever been identified as the source of an LD outbreak.

The retrospective identification of a cluster of four cases that occurred in January 2019, including two for which the Lp 1 ST62 strain was linked to the environmental strain in the November 2019 outbreak, may indicate previous contamination of the heating plant. It cannot be ruled out that other cases of LD that occurred between November 2017 and 12 December 2019 and that had been in the outbreak area during their incubation period are due to this boiler room because no WGS analysis was possible on these cases due to the absence of a clinical strain. However, no cluster of cases, suggesting the presence of a common source of contamination, was detected before 2019. In addition, an active case search was carried out during the November 2019 outbreak investigation to ensure there were no undeclared cases The high temperatures recorded in the summer of 2019 [25], might explain the high level of *Legionella* contamination of the condenser as soon as the plant was turned on in October 2019 for the winter season.

In the past, major LD outbreaks have had a significant impact on public health [12,13,15,30,31, 32]. In the case of the 2019 Strasbourg outbreak, effective coordination of rapid epidemiological, environmental and microbiological investigations contributed to the rapid identification of the collective biomass heating plant as the most likely, albeit unusual, source of contamination. Given a context where the number of collective biomass heating systems in France is increasing as an alternative to fossil fuel processes, and as the extent of the deployment of these systems is not known particularly in Europe, it is important that these facilities be considered by health authorities as potential sources of contamination in LD cluster investigations. In a wider context, our findings suggest technological advances to address climate change may promote the proliferation and spread of Lp. Moreover, in a context where the European population is ageing, outbreaks of LD could become serious public health issues [33].

A limitation of this study is that not all 28 LD cases included in this episode could be individually linked to the source, because some of them lacked or had only partial microbiological data. On the other hand, all individual sources that could have potentially been contaminated were investigated. *Legionella* contamination in individual installations was found for two cases (both home water networks), but the strains identified there were of a different serotype to those found in outbreak cases. This points, together with other available evidence, to consider the biomass condensing boiler as the source of contamination for the outbreak.

## Conclusion

This study highlights the importance of conducting prompt field investigations after LD outbreaks have been notified, and persisting, when no source has rapidly been found, to identify prior undetected sources of contamination. Regarding the plant in Strasbourg, the boiler at the source of the 2019 outbreak was restarted in October 2020 after comprehensive measures to improve safety were implemented. Following the 2019 Strasbourg outbreak, the French Ministry of the Environment mandated a full risk analysis of biomass heating systems similar to the one identified as the outbreak source of contamination. Moreover, French national regulations were improved in January 2021 to include the risks associated with this type of process [34].

## Data Availability

Raw reads were deposited on ENA database under the study accession number PRJEB98926.

## Supplementary Data

833000 SupplementaryMaterial

## References

[1] Phin N Parry-Ford F Harrison T Stagg HR Zhang N Kumar K Epidemiology and clinical management of Legionnaires’ disease. Lancet Infect Dis. 2014;14(10):1011-21. 10.1016/S1473-3099(14)70713-3 24970283

[2] European Centre for Disease Prevention and Control (ECDC). Legionnaires’ disease. Annual Epidemiological Report for 2021. Stockholm: ECDC; 2023.

[3] Mondino S Schmidt S Rolando M Escoll P Gomez-Valero L Buchrieser C. Legionnaires’ Disease: State of the Art Knowledge of Pathogenesis Mechanisms of *Legionella.* Annu Rev Pathol. 2020;15(1):439-66. 10.1146/annurev-pathmechdis-012419-032742 31657966

[4] Borges V Nunes A Sampaio DA Vieira L Machado J Simões MJ Legionella pneumophila strain associated with the first evidence of person-to-person transmission of Legionnaires’ disease: a unique mosaic genetic backbone. Sci Rep. 2016;6(1):26261. 10.1038/srep26261 27196677 PMC4872527

[5] Fisman DN Lim S Wellenius GA Johnson C Britz P Gaskins M It’s not the heat, it’s the humidity: wet weather increases legionellosis risk in the greater Philadelphia metropolitan area. J Infect Dis. 2005;192(12):2066-73. 10.1086/498248 16288369

[6] Ng V Tang P Jamieson F Drews SJ Brown S Low DE Going with the flow: legionellosis risk in Toronto, Canada is strongly associated with local watershed hydrology. EcoHealth. 2008;5(4):482-90. 10.1007/s10393-009-0218-0 19370300

[7] Pampaka D Gómez-Barroso D López-Perea N Carmona R Portero RC. Meteorological conditions and Legionnaires’ disease sporadic cases-a systematic review. Environ Res. 2022;214(Pt 4):114080. 10.1016/j.envres.2022.114080 35964674

[8] Hicks LA Rose CE Jr Fields BS Drees ML Engel JP Jenkins PR Increased rainfall is associated with increased risk for legionellosis. Epidemiol Infect. 2007;135(5):811-7. 10.1017/S0950268806007552 17121693 PMC2870637

[9] Den Boer JW Yzerman EPF Schellekens J Lettinga KD Boshuizen HC Van Steenbergen JE A large outbreak of Legionnaires’ disease at a flower show, the Netherlands, 1999. Emerg Infect Dis. 2002;8(1):37-43. 10.3201/eid0801.010176 11749746 PMC2730281

[10] Hammami N Laisnez V Wybo I Uvijn D Broucke C Van Damme A A cluster of Legionnaires’ disease in Belgium linked to a cooling tower, August-September 2016: practical approach and challenges. Epidemiol Infect. 2019;147:e326. 10.1017/S0950268819001821 31858932 PMC7006017

[11] Hlavsa MC Cikesh BL Roberts VA Kahler AM Vigar M Hilborn ED Outbreaks Associated with Treated Recreational Water - United States, 2000-2014. MMWR Morb Mortal Wkly Rep. 2018;67(19):547-51. 10.15585/mmwr.mm6719a3 29771872 PMC6048947

[12] Loenenbach AD Beulens C Euser SM van Leuken JPG Bom B van der Hoek W Two Community Clusters of Legionnaires’ Disease Directly Linked to a Biologic Wastewater Treatment Plant, the Netherlands. Emerg Infect Dis. 2018;24(10):1914-8. 10.3201/eid2410.180906 30226165 PMC6154163

[13] Ricci ML Rota MC Caporali MG Girolamo A Scaturro M. A Legionnaires’ Disease Cluster in a Private Building in Italy. Int J Environ Res Public Health. 2021;18(13):6922. 10.3390/ijerph18136922 34203343 PMC8297097

[14] Ryu S Yang K Chun BC. Community-acquired Legionnaires’ Disease in a Newly Constructed Apartment Building. J Prev Med Public Health. 2017;50(4):274-7. 10.3961/jpmph.17.066 28768406 PMC5541279

[15] Nhu Nguyen TM Ilef D Jarraud S Rouil L Campese C Che D A community-wide outbreak of legionnaires disease linked to industrial cooling towers--how far can contaminated aerosols spread? J Infect Dis. 2006;193(1):102-11. 10.1086/498575 16323138

[16] Cebrián F Montero JC Fernández PJ. New approach to environmental investigation of an explosive legionnaires´ disease outbreak in Spain: early identification of potential risk sources by rapid Legionella spp immunosensing technique. BMC Infect Dis. 2018;18(1):696. 10.1186/s12879-018-3605-8 30587144 PMC6307211

[17] Santé publique France 2020 (SPF). Quels sont les risques liés à la pollution de l'eau? [What are the risks related to water pollution?]. Saint Maurice: SPF; Updated on 20 Jun 2023. French. Available from: https://www.santepubliquefrance.fr/determinants-de-sante/pollution-et-sante/eau/les-enjeux-de-sante/quels-sont-les-risques-lies-a-la-pollution-de-l-eau#:~:text=L%E2%80%99Agence%20nationale%20de%20s%C3%A9curit%C3%A9%20sanitaire%20de%20l%27alimentation%2C%20de,param%C3%A8tres%20inscrits%20au%20code%20de%20la%20sant%C3%A9%20publique

[18] Santé publique France (SPF). Bilan des cas de légionellose survenus en France en 2019. [Report on cases of Legionnaires' disease in France in 2019]. Saint Maurice: SPF; 2020. French. Available from: https://www.santepubliquefrance.fr/maladies-et-traumatismes/maladies-et-infections-respiratoires/legionellose/articles/bilan-des-cas-de-legionellose-survenus-en-france-en-2019

[19] Campèse C Descours G Lepoutre A Beraud L Maine C Che D Legionnaires’ disease in France. Med Mal Infect. 2015;45(3):65-71. 10.1016/j.medmal.2015.01.015 25722040

[20] Rousseau C Ginevra C Simac L Fiard N Vilhes K Ranc AG A Community Outbreak of Legionnaires’ Disease with Two Strains of *L. pneumophila* Serogroup 1 Linked to an Aquatic Therapy Centre. Int J Environ Res Public Health. 2022;19(3):1119. 10.3390/ijerph19031119 35162143 PMC8834728

[21] Haut Conseil de la Santé publique (HCSP). Risque lié aux légionelles Guide d'investigation et d'aide à la gestion. [Legionella related risk: investigation guidance and management support]. Paris: HCSP; 2013.

[22] KoSamti. Expertise chaufferie biomasse. Rapport d'expertise. [Biomass boiler room expertise. Expert report]. 13 Mar 2020. French.

[23] Wick RR Judd LM Gorrie CL Holt KE. Unicycler: Resolving bacterial genome assemblies from short and long sequencing reads. PLOS Comput Biol. 2017;13(6):e1005595. 10.1371/journal.pcbi.1005595 28594827 PMC5481147

[24] Ginevra C Lopez M Forey F Reyrolle M Meugnier H Vandenesch F Evaluation of a nested-PCR-derived sequence-based typing method applied directly to respiratory samples from patients with Legionnaires’ disease. J Clin Microbiol. 2009;47(4):981-7. 10.1128/JCM.02071-08 19225096 PMC2668346

[25] Météo-France. 2019 au 3e rang des années les plus chaudes. [2019 ranks third among the warmest years recorded]. Bilan de l'année 2019: s'est caractérisée par un soleil généreux et la prédominance de la douceur tout au long de l'année avec deux vagues de chaleur d'une intensité exceptionnelle durant l'été. French. Paris: Météo-France; 2019. [Updated 01/10/2020]. Available from: https://meteofrance.com/actualites/climat/france-2019-au-3e-rang-des-annees-les-plus-chaudes.

[26] Anacarso I Guerrieri E Bondi M de Niederhäusern S Iseppi R Sabia C Influence of Legionella pneumophila and other water bacteria on the survival and growth of Acanthamoeba polyphaga. Arch Microbiol. 2010;192(10):877-82. 10.1007/s00203-010-0618-0 20730523

[27] Walser SM Gerstner DG Brenner B Höller C Liebl B Herr CE. Assessing the environmental health relevance of cooling towers--a systematic review of legionellosis outbreaks. Int J Hyg Environ Health. 2014;217(2-3):145-54. 10.1016/j.ijheh.2013.08.002 24100053

[28] Vermeulen LC Brandsema PS van de Kassteele J Bom BCJ Sterk HAM Sauter FJ Atmospheric dispersion and transmission of Legionella from wastewater treatment plants: A 6-year case-control study. Int J Hyg Environ Health. 2021;237:113811. 10.1016/j.ijheh.2021.113811 34311418

[29] Villanueva D Schepanski K. Investigation of atmospheric conditions fostering the spreading of legionnaires’ disease in outbreaks related to cooling towers. Int J Biometeorol. 2019;63(10):1347-56. 10.1007/s00484-019-01751-9 31342243

[30] Shivaji T Sousa Pinto C San-Bento A Oliveira Serra LA Valente J Machado J A large community outbreak of Legionnaires disease in Vila Franca de Xira, Portugal, October to November 2014. Euro Surveill. 2014;19(50):20991. 10.2807/1560-7917.ES2014.19.50.20991 25597540

[31] García-Fulgueiras A Navarro C Fenoll D García J González-Diego P Jiménez-Buñuales T Legionnaires’ disease outbreak in Murcia, Spain. Emerg Infect Dis. 2003;9(8):915-21. 10.3201/eid0908.030337 12967487 PMC3020623

[32] Faccini M Russo AG Bonini M Tunesi S Murtas R Sandrini M Large community-acquired Legionnaires’ disease outbreak caused by *Legionella pneumophila* serogroup 1, Italy, July to August 2018. Euro Surveill. 2020;25(20):1900523. 10.2807/1560-7917.ES.2020.25.20.1900523 32458793 PMC7262491

[33] World Health Organization (WHO). Disease Outbreak News; Legionellosis in Poland. Geneva: WHO; 14 September 2023. Available from: https://www.who.int/emergencies/disease-outbreak-news/item/2023-DON487

[34] Arrêté du 23 juillet 2021 modifiant l'arrêté du 14 décembre 2013 relatif aux prescriptions générales applicables aux installations relevant du régime de la déclaration au titre de la rubrique n° 2921 de la nomenclature des installations classées pour la protection de l'environnement [Order of July 23 2021 amending the order of December 14 2013 relating to the general requirements applicable to facilities falling under the declaration regime under heading No. 2921 of the nomenclature of classified facilities for environmental protection]. NOR : TREP2014720A ELI. JORF n°0171 du 25 juillet 2021.Texte n° 6. French. Available from: https://www.legifrance.gouv.fr/eli/arrete/2021/7/23/TREP2014720A/jo/texte

