# Supplementary material for: A community Legionnaires’ disease outbreak linked to a collective biomass condensing boiler, France, 2019
Source: Euro Surveill. 2025 Oct 16;30(41):2400804. doi: 10.2807/1560-7917.ES.2025.30.41.2400804 (PMC12534781; doi:10.2807/1560-7917.ES.2025.30.41.2400804)
Supplement: SupplementaryMaterial [file 24-00804_SupplementaryFigureS1.pdf]

This supplementary material is hosted by Eurosurveillance as supporting information alongside the article "**A community Legionnaires' disease outbreak linked to a collective biomass condensing boiler, France, 2019**", on behalf of the authors, who remain responsible for the accuracy and appropriateness of the content. The same standards for ethics, copyright, attributions and permissions as for the article apply. Supplements are not edited by Eurosurveillance and the journal is not responsible for the maintenance of any links or email addresses provided therein

METEO FRANCE

ROSE DES VENTS

Vent horaire à 10 mètres, moyenné sur 10 mn

Année 2019 – Du 24 OCTOBRE au 12 DÉCEMBRE

STRASBOURG-ENTZHEIM (67)

Indicatif : 67124001, alt : 150 m., lat : 48°32'58"N, lon : 7°38'25"E

Fréquence des vents en fonction de leur provenance en %

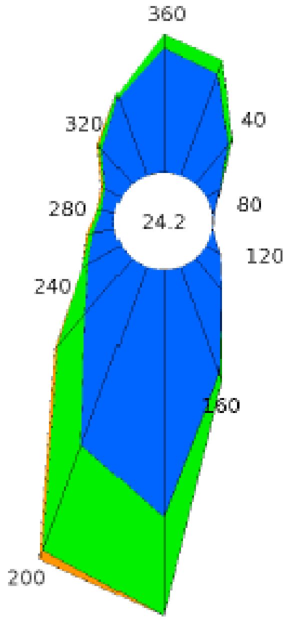

Valeurs horaires entre 0h00 et 23h00, heure UTC

Tableau de répartition
Nombre de cas étudiés : 1200
Manquants : 0

Dir.	[1.5;4.5 [	[4.5;8.0]	> 8.0 m/s	Total
20	5.3	0.8	0.0	6.0
40	2.5	0.3	0.0	2.8
60	0.8	+	0.0	0.8
80	0.1	0.0	0.0	0.1
100	+	0.0	0.0	+
120	0.8	0.0	0.0	0.8
140	2.0	0.0	0.0	2.0
160	5.5	0.4	0.0	6.0
180	12.2	4.8	0.0	17.0
200	9.3	5.5	0.5	15.3
220	3.5	2.0	0.3	5.8
240	1.8	0.3	0.0	2.1
260	1.0	0.2	0.1	1.4
280	0.6	0.2	+	0.8
300	0.8	+	0.1	1.0
320	2.2	0.3	0.1	2.6
340	4.1	0.2	0.0	4.3
360	6.1	0.7	0.0	6.8
Total	58.8	15.8	1.2	75.8
[0;1.5 [				24.2

Groupes de vitesses (m/s)
[1.5;4.5 [[4.5;8.0] > à 8.0

Pourcentage par direction
0% 5% 10%

Dir. : Direction d'où vient le vent en rose de 360° : 90° = Est, 180° = Sud, 270° = Ouest, 360° = Nord
le signe + indique une fréquence non nulle mais inférieure à 0.1%

Edité le : 28/01/2020 dans l'état de la base

N.B. : La vente, redistribution ou rediffusion des informations reçues, en l'état ou sous forme de produits dérivés, est strictement interdite sans l'accord de METEO-FRANCE

METEO-FRANCE Nord-Est – Division des Etudes
Parc d'Innovation – Bld G. d'Andernach – BP 50120 67403 Illkirch Cedex
Tél. : 03 88 40 42 56 – Fax : 03 88 40 42 10 – Email : etudes_clim.nord-est@meteo.fr
